# Quality‐Adjusted Time Without Symptoms of Disease or Toxicity (Q‐TWiST) in Patients With Newly Diagnosed Philadelphia Chromosome‐Positive Acute Lymphoblastic Leukemia: A Comparison of Ponatinib Versus Imatinib

**DOI:** 10.1002/cam4.70780

**Published:** 2025-03-31

**Authors:** Ajibade Ashaye, Ling Shi, Ibrahim Aldoss, Pau Montesinos, Pankit Vachhani, Vanderson Rocha, Cristina Papayannidis, Jessica T. Leonard, Maria R. Baer, Jose‐Maria Ribera, James McCloskey, Jianxiang Wang, Deepali Rane, Shien Guo

**Affiliations:** ^1^ Takeda Development Center Americas Inc. Cambridge Massachusetts USA; ^2^ Evidera Inc. Bethesda Maryland USA; ^3^ City of Hope National Medical Center Duarte California USA; ^4^ Hospital Universitari i Politècnic La Fe Valencia Spain; ^5^ University of Alabama at Birmingham Birmingham Alabama USA; ^6^ Instituto Do Câncer Do Estado de São Paulo São Paulo Brazil; ^7^ IRCCS Azienda Ospedaliero‐Universitaria di Bologna, Istituto di Ematologia “L. e A. Seràgnoli,” Bologna Italy; ^8^ Oregon Health and Science University Portland Oregon USA; ^9^ University of Maryland Marlene and Stewart Greenebaum Comprehensive Cancer Center Baltimore Maryland USA; ^10^ ICO—Hospital Germans Trias i Pujol, Josep Carreras Leukaemia Research Institute Badalona Spain; ^11^ Hackensack University Medical Center Hackensack New Jersey USA; ^12^ Institute of Hematology & Blood Diseases Hospital of CAMS & PUMC Tianjin China

**Keywords:** acute lymphoblastic leukemia, imatinib, Philadelphia chromosome‐positive, ponatinib, Q‐TWiST analysis, quality of life, tyrosine kinase inhibitor

## Abstract

**Background:**

In the phase 3 ponatinib‐3001 trial (PhALLCON, NCT03589326), ponatinib demonstrated superior efficacy over imatinib with comparable safety in patients with newly diagnosed Philadelphia‐positive acute lymphoblastic leukemia (Ph+ ALL). This post hoc analysis evaluated the net benefits of ponatinib using a quality‐adjusted time without symptoms of disease or toxicity (Q‐TWiST) approach.

**Methods:**

Overall survival (OS) time for patients from PhALLCON was partitioned into three health states: TOX (time with grade 3+ treatment‐emergent adverse events [TEAEs] before disease progression), TWiST (time without toxicity before progression), and REL (time from progression until death or end of follow‐up). Q‐TWiST was calculated as the sum of health utility‐weighted restricted mean durations of the three states. A relative Q‐TWiST gain of ≥ 10% was considered clinically important. Sensitivity analyses were conducted by varying TOX and REL utilities, follow‐up time, and the TOX definition (using grade 2+ TEAEs or patient‐perceived treatment tolerability assessed by the FACT‐GP5).

**Results:**

Among all randomized patients (ponatinib *n* = 164, imatinib *n* = 81), restricted mean OS was similar between arms (1082.2 vs. 1024.8 days; *p* = 0.373). In the base‐case analysis, mean TWiST was 214.5 days longer with ponatinib versus imatinib (95% CI 70.3–358.7; *p* = 0.004), REL was shorter by 175.9 days (325.4–26.5; *p* = 0.021), and TOX was not significantly different between arms (*p* = 0.228). The relative Q‐TWiST gain (10.98%) was clinically important. Sensitivity analyses consistently supported the robustness of the base‐case findings.

**Conclusion:**

Ponatinib may prolong quality‐adjusted survival compared with imatinib, supporting the benefit–risk profile of ponatinib as a front‐line treatment for Ph+ ALL.

**Trial Registration:**

NCT03589326

## 1 Introduction

Philadelphia chromosome‐positive (Ph+) acute lymphoblastic leukemia (ALL) is a subtype of ALL that carries the *BCR::ABL1* fusion gene, which encodes the constitutively active BCR::ABL oncoprotein [1]. Ph+ ALL accounts for approximately 20%–25% of ALL cases in adults and 3%–5% in children [2, 3]. Prior to the advent of tyrosine kinase inhibitors (TKIs), which block the activity of the BCR::ABL oncoprotein, Ph+ ALL was treated with chemotherapy alone, and the prognosis was poor [4]. The addition of a TKI to chemotherapy improved the rates of complete remission (CR) and overall survival (OS) in patients with Ph+ ALL [4]. The combination of chemotherapy and a TKI has become the standard of care for newly diagnosed Ph+ ALL [5].

Ponatinib is a third‐generation TKI that effectively inhibits both unmutated and mutated BCR::ABL1 [6, 7]. Ponatinib with chemotherapy received accelerated approval from the US Food and Drug Administration on March 19, 2024, for adults with newly diagnosed Ph+ ALL [8]. In the phase 3 ponatinib‐3001 trial (NCT03589326, hereafter referred to as PhALLCON), the first study directly comparing two TKIs for the treatment of Ph+ ALL in the front‐line setting, the rate of minimal residual disease (MRD)‐negative CR at the end of induction (primary endpoint) was higher with ponatinib than imatinib (34% vs. 17%; risk difference [95% CI] 0.18 [0.06‐0.29]) [9]. Median progression‐free survival (PFS) was longer with ponatinib than imatinib (20.0 vs. 7.9 months; hazard ratio [95% CI] 0.58 [0.41–0.83]). The rates of grade 3–4 treatment‐emergent adverse events (TEAEs) were similar in the two arms (85% vs. 88%). At the data cutoff (August 12, 2022), median OS was not reached in either arm. The difference in OS between treatments was therefore not evaluated due to data immaturity.

Reflecting the better treatment effect and similar safety profile, patients in PhALLCON receiving ponatinib stayed on treatment longer than those receiving imatinib. The median time to discontinuation due to toxicity or lack of efficacy was 22 months in the imatinib arm but was not reached in the ponatinib arm [9]. Due to this longer exposure, patients in the ponatinib arm may have experienced more TEAEs during treatment, which can lead to a worse quality of life [10, 11]. Further assessment is therefore necessary to better understand the potential trade‐offs between the PFS benefits associated with ponatinib and the possible impact on quality of life. This assessment would be expected to provide further details on the net clinical benefits of ponatinib and help guide clinicians and patients in making informed treatment decisions.

The quality‐adjusted time without symptoms of disease or toxicity (Q‐TWiST) method provides a single metric to compare net clinical benefits between treatments by accounting for both the duration and quality of survival [12, 13]. In a Q‐TWiST analysis, a patient's survival time is partitioned into distinct health states according to the status of treatment toxicities and disease progression. These health states are then weighted by patient preferences, which are measured by health utilities and indicate how much a patient values a given health state or outcome [14, 15].

This current analysis used the Q‐TWiST method to evaluate whether the longer PFS with ponatinib versus imatinib in PhALLCON came at the cost of a worse quality of life.

## 2 Materials and Methods

### 2.1 Study Design and Participants

This is a post hoc analysis using data from the phase 3 PhALLCON trial (data cutoff August 12, 2022). Details of patient eligibility and the trial design have been reported previously [9]. The trial was conducted in accordance with the International Council for Harmonization Guideline for Good Clinical Practice and the principles of the Declaration of Helsinki. The study protocol and all amendments were approved by the independent institutional review board or the independent ethics committee at each study site. All patients provided written informed consent prior to study participation.

Patients (age ≥ 18 years) with newly diagnosed Ph+ ALL and an Eastern Cooperative Oncology Group performance status of ≤ 2 were randomized 2:1 to receive oral ponatinib (30 mg) or imatinib (600 mg) once daily in combination with reduced‐intensity chemotherapy through induction (3 cycles), consolidation (6 cycles), and maintenance (11 cycles) in 28‐day cycles. The ponatinib dose was reduced from 30 mg/day to 15 mg/day if a patient achieved MRD‐negative CR at or after the end of induction. After cycle 20, patients received single‐agent ponatinib or imatinib until disease progression, relapse from CR, unacceptable toxicity, receipt of a hematopoietic stem cell transplant or an alternative therapy, or withdrawal of consent. All randomized patients were included in the current analysis.

In PhALLCON, OS was defined as the time from randomization to death due to any cause. PFS was a secondary endpoint defined as death due to any cause, failure to achieve CR by the end of induction, failure to achieve MRD negativity by the end of treatment, or loss of MRD negativity, and was evaluated in a post hoc analysis. Disease progression was defined as an increase of ≥ 25% in the absolute number of circulating or bone marrow blasts or the development of extramedullary disease. Safety outcomes included TEAEs, which were graded according to the Common Terminology Criteria for Adverse Events, version 5.0 [16].

Patient‐reported outcomes (PROs) were included as exploratory endpoints in PhALLCON. The present analysis considered one of these PROs: item GP5 of the patient‐reported Functional Assessment of Cancer Therapy–Leukemia (FACT‐GP5). Using the question “I am bothered by side effects of treatment,” FACT‐GP5 assesses overall treatment tolerability from the patient's perspective [17]. FACT‐GP5 response options comprise five levels of severity: “not at all”, “a little bit”, “somewhat”, “quite a bit”, and “very much”. PROs were assessed at baseline; day 1 of cycles 1, 4, 7, 10, 13, 16, 19, and 21, and at least every 6 cycles thereafter; and at the end of treatment.

### 2.2 Q‐TWiST Analysis

#### 2.2.1 Q‐TWiST Method

Patients' survival time in the study was partitioned into three mutually exclusive health states: toxicity (TOX), time without symptoms or toxicities (TWiST), and relapse (REL). TOX was the time with toxicity after randomization and before disease progression. The number of days with toxicity was summed for each patient up to the earliest of toxicity resolution, disease progression, death, or end of follow‐up. A day with multiple TEAEs was only counted once. TWiST was the time in the progression‐free period without toxicity, calculated as the duration of PFS minus that of TOX. REL was the time from disease progression until death or the end of follow‐up, whichever occurred first, and was calculated as the duration of OS minus that of PFS.

#### 2.2.2 Base‐Case Analysis

In the base‐case analysis, toxicity was defined as grade 3+ TEAEs because they were more likely to have a greater impact on patients' quality of life than those of grade < 3. TOX was 0 for patients without any grade 3 #x0002B; TEAEs. Follow‐up was the maximum OS follow‐up time observed at the data cutoff (1247 days). Kaplan–Meier estimates of TOX, PFS, and OS were used to generate partitioned survival plots depicting the TOX, TWiST, and REL states. Restricted mean survival time, defined as the mean area under the survival curve up to a specific time point, was estimated for PFS, OS, and each health state up to the maximum follow‐up time; 95% confidence intervals (CIs) were estimated using a non‐parametric bootstrapping approach with replacement (1000 replications).

Q‐TWiST was calculated as the sum of utility‐weighted restricted mean durations of the three health states: $Q-\text{TWiST}=u_{\mathrm{TOX}}\times \mathrm{TOX}+u_{\text{TWiST}}\times \text{TWiST}+u_{\mathrm{REL}}\times \mathrm{REL}$, where *u*_*x*_ is the average utility for health state *x* [18]. Health state utilities from previous studies in similar patient populations [19, 20, 21] were used. A health state utility typically ranges from 0 (death) to 1 (“perfect” health). In the current analysis, a utility value of 0.80 was used for TWiST; the TOX utility was assumed to be 0.60, a 0.20 decrement from the TWiST utility, and the REL utility was set at 0.40, a 0.40 decrement from that of TWiST. Relative Q‐TWiST gains were calculated as the difference in Q‐TWiST between the ponatinib and imatinib arms (absolute Q‐TWiST gain), divided by the restricted mean OS of the imatinib arm. A relative Q‐TWiST gain of ≥ 10% was considered clinically important [22].

#### 2.2.3 Threshold Analysis

Health utility values can differ among individuals because they might have varying perceptions regarding the impact of treatment toxicities or disease progression on their quality of life. To understand how the Q‐TWiST gain might change with individual preferences and to examine the robustness of the base‐case analysis results, a two‐way threshold analysis was conducted by varying the utility values of TOX and REL between 0 and 1. The TWiST utility was fixed at 0.80, as in the base‐case analysis.

#### 2.2.4 Sensitivity Analyses

To understand the impact of follow‐up duration on Q‐TWiST, a sensitivity analysis was conducted to estimate Q‐TWiST at various follow‐up times. Between‐arm differences in Q‐TWiST were calculated by incrementally varying the follow‐up time in 4‐week intervals until the maximum follow‐up time was reached. Another sensitivity analysis was conducted to re‐evaluate Q‐TWiST with the TOX state defined as grade 2+ TEAEs, as lower‐grade TEAEs may also have an impact on quality of life.

To better understand the impact of patient‐perceived overall treatment tolerability on Q‐TWiST, a further sensitivity analysis was conducted by defining the TOX state as a FACT‐GP5 response of “quite a bit” or “very much”. Given that FACT‐GP5 was assessed per a fixed schedule, the start date of TOX was the date of the earliest assessment visit before disease progression with a response of “quite a bit” or “very much”. The end date of TOX was the earliest of the following: the first visit when the response level decreased to “not at all”, “a little bit**”**, or “somewhat”; disease progression; death; or the end of follow‐up. TOX was set to 0 for patients without any FACT‐GP5 assessments during the study. Missing data were handled by last observation carried forward.

For all sensitivity analyses, the utility value for each health state was the same as in the base‐case analysis.

#### 2.2.5 Statistical Analysis

Q‐TWiST analyses were performed using the “qtwist” function of the R package (version 4.3.1, R Foundation, Vienna, Austria) [18]. Consistent with previous literature of Q‐TWiST analyses [13, 18, 23, 24], nominal *p*‐values were estimated without adjustment for multiple comparisons due to the exploratory nature of the outcomes [25].

## 3 Results

### 3.1 Base‐Case Analysis

The Q‐TWiST analysis included 164 patients randomized to ponatinib and 81 randomized to imatinib. Restricted mean OS (95% CI) was 1082.2 days (1018.8–1145.7) for the ponatinib arm and 1024.8 days (918.7–1131.0) for the imatinib arm (*p* = 0.373) (Table 1). Restricted mean PFS (95% CI) was significantly longer with ponatinib (684.5 days [590.2–778.9]) than with imatinib (451.2 days [338.9–563.5]; *p* = 0.002).

**TABLE 1:** Restricted mean duration of each health state and Q‐TWiST (base‐case analysis).

States	Mean (95% CI) duration, days		Difference in means (95% CI), days (ponatinib vs. imatinib)	*p*
	Ponatinib (*n* = 164)	Imatinib (*n* = 81)		
OS	1082.2 (1018.8, 1145.7)	1024.8 (918.7, 1131.0)	57.4 (−68.9, 183.7)	0.373
PFS	684.5 (590.2, 778.9)	451.2 (338.9, 563.5)	233.3 (87.4, 379.2)	0.002
TOX	96.2 (74.9, 117.5)	77.3 (55.0, 99.6)	18.9 (−11.8, 49.5)	0.228
TWiST	588.3 (494.5, 682.2)	373.9 (262.4, 485.4)	214.5 (70.3, 358.7)	0.004
REL	397.7 (306.5, 488.9)	573.6 (452.4, 694.8)	−175.9 (−325.4, −26.5)	0.021
Q‐TWiST	687.5 (634.9, 740.1)	575.0 (502.7, 647.2)	112.5 (21.9, 203.1)	0.015

*Note:* Data are restricted means at the maximum follow‐up time (1247 days). Utility values of 0.8 for TWiST, 0.6 for TOX, and 0.4 for REL were used for TWiST estimation. TOX was defined as grade 3+ TEAEs.

Abbreviations: CI, confidence interval; OS, overall survival; PFS, progression‐free survival; Q‐TWiST, quality‐adjusted TWiST; REL, relapse (period from disease progression until end of follow‐up/death); TEAE, treatment‐emergent adverse event; TOX, toxicity (sum of all periods in which patients experienced grade 3 + TEAEs during the progression‐free period); TWiST, time without symptoms or toxicities.

**FIGURE 1:**
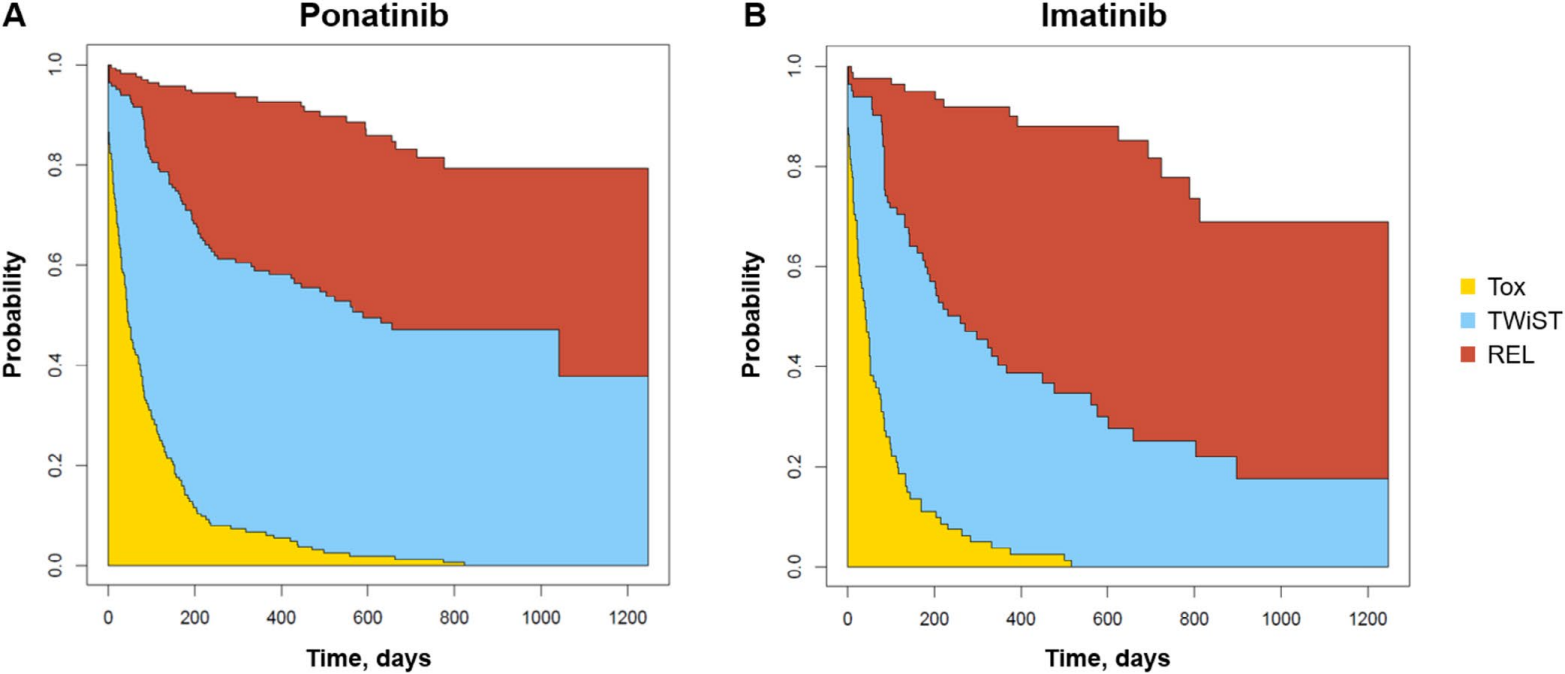
Partitioned survival curves in the base‐case analysis. (A) Ponatinib. (B) Imatinib. Abbreviations: REL, relapse (period from disease progression until end of follow‐up/death); TEAE, treatment‐emergent adverse event; TOX, toxicity (sum of all periods in which patients experienced grade 3+ TEAEs during the progression‐free period); TWiST, time without symptoms or toxicities.

Mean TWiST was longer with ponatinib than imatinib by 214.5 days (95% CI 70.3–358.7; *p* = 0.004), and REL was shorter by 175.9 days (325.4–26.5; *p* = 0.021) (Table 1; Figure 1). TOX was not significantly different between the two arms (*p* = 0.228). Mean Q‐TWiST was longer with ponatinib than imatinib by 112.5 days (95% CI 21.9–203.1; *p* = 0.015) (Table 1). The relative Q‐TWiST gain was 10.98%, exceeding the pre‐specified threshold of 10% for clinical meaningfulness.

### 3.2 Threshold Analysis

**FIGURE 2:**
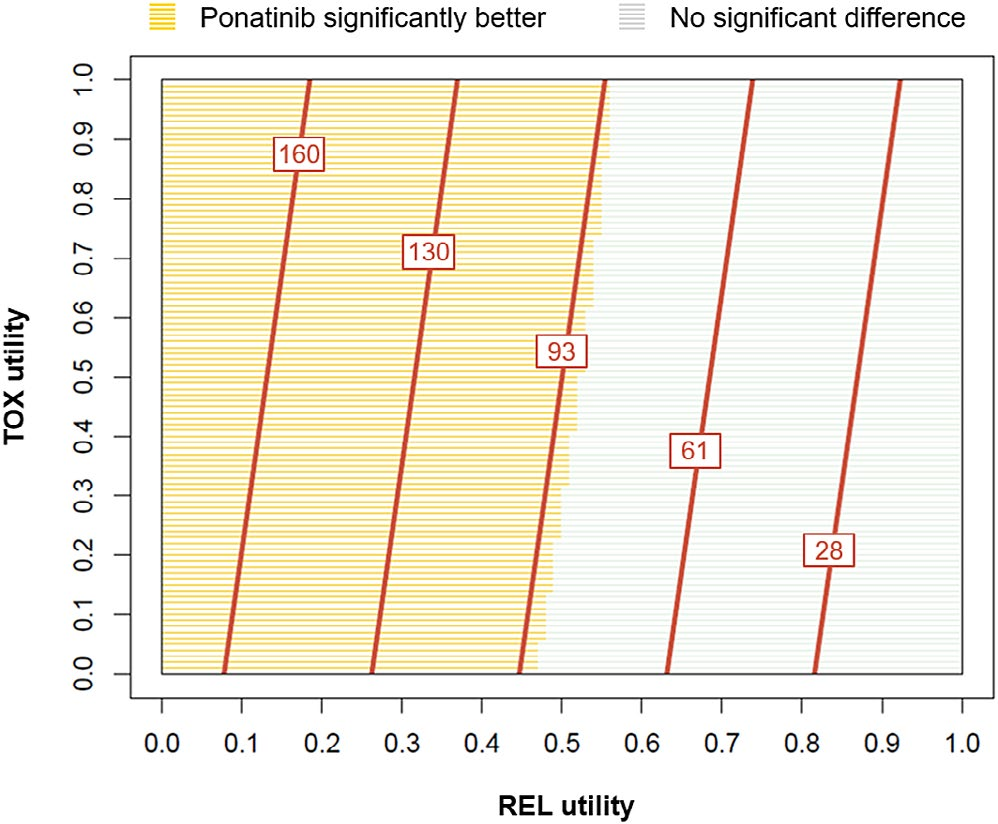
Threshold utility plot of absolute Q‐TWiST gain (in days) with ponatinib relative to imatinib at the maximum follow‐up of 1247 days. The TOX and REL utility values varied from 0 to 1, while that of TWiST was fixed at 0.8. Red lines represent the average Q‐TWiST gain (in days) with ponatinib compared to imatinib, and numbers associated with the red lines are the absolute Q‐TWiST gain for the corresponding utility values. The color of the shaded horizontal lines represents the significance of the comparison, with yellow indicating a significantly better effect (*p* < 0.05) with ponatinib than imatinib and light gray indicating no significant difference in treatment effect. Abbreviations: Q‐TWiST, quality‐adjusted TWiST; REL, relapse (period from disease progression until end of follow‐up/death); TEAE, treatment‐emergent adverse event; TOX, toxicity (sum of all periods in which patients experienced grade 3+ TEAEs during the progression‐free period); TWiST, time without symptoms or toxicities.

In the threshold analysis, the Q‐TWiST gains with ponatinib relative to imatinib were positive for all possible combinations of TOX and REL utility values (Figure 2); that is, the ponatinib arm had a numerically greater Q‐TWiST than imatinib regardless of TOX and REL utility values. The Q‐TWiST gains were statistically significant for all TOX utility values examined when the REL utility was below approximately 0.47 (i.e., the area with yellow shaded lines in Figure 2).

### 3.3 Sensitivity Analyses

**FIGURE 3:**
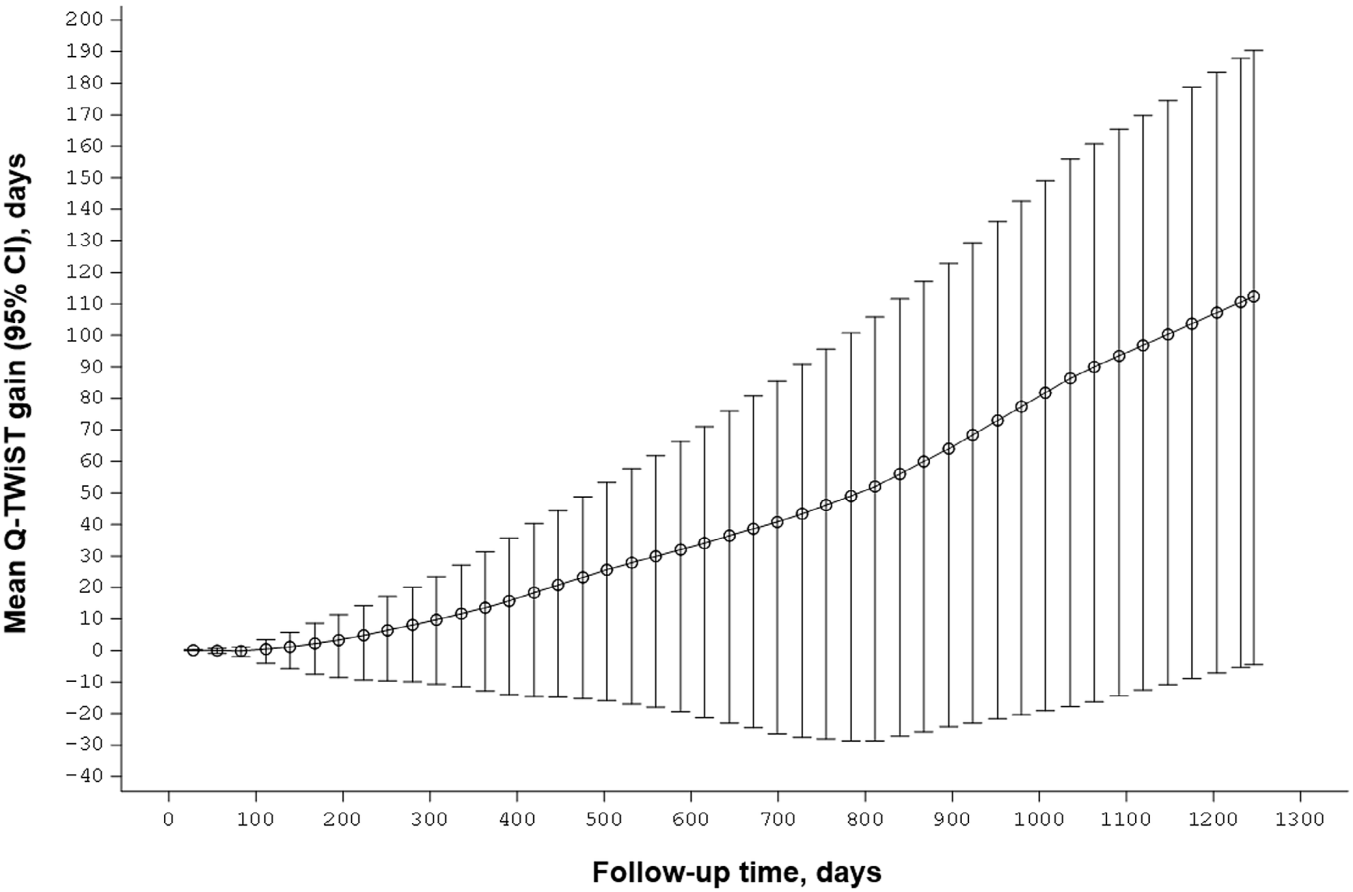
Absolute Q‐TWiST gain (in days) with ponatinib relative to imatinib at various follow‐up times. The Q‐TWiST gain was estimated using the utility values in the base‐case analysis. Error bars represent the 95% CI. Abbreviations: CI, confidence interval; Q‐TWiST, quality‐adjusted TWiST; TWiST, time without symptoms or toxicities.

The absolute Q‐TWiST gain with ponatinib compared with imatinib (i.e., the mean Q‐TWiST gain in days) increased over time and was highest at the maximum follow‐up of 1247 days (Figure 3).

In the sensitivity analysis using grade 2+ TEAEs to define the TOX state, TWiST was longer with ponatinib than with imatinib by 190.2 days (95% CI 64.2–316.2; *p* = 0.003) (Table 2; Figure S1). REL was shorter with ponatinib than with imatinib by 175.9 days (95% CI 324.9–27.0; *p* = 0.021). No significant difference was found in TOX between the two arms (*p* = 0.131). The absolute Q‐TWiST gain with ponatinib relative to imatinib was 107.7 days (95% CI 24.9–190.4; *p* = 0.011), corresponding to a relative Q‐TWiST gain of 10.50%.

**TABLE 2:** Restricted mean duration of each health state and Q‐TWiST with the TOX state defined by grade 2+ TEAEs (sensitivity analysis).

States	Mean (95% CI) duration, days		Difference in means (95% CI), days (ponatinib vs. imatinib)	*p*
	Ponatinib (*n* = 164)	Imatinib (*n* = 81)		
OS	1082.2 (1017.3, 1147.1)	1024.8 (918.6, 1131.1)	57.4 (−66.8, 181.6)	0.365
PFS	684.5 (593.4, 775.6)	451.2 (343.6, 558.9)	233.3 (94.3, 372.3)	0.001
TOX	212.8 (177.2, 248.4)	169.7 (125.1, 214.2)	43.2 (−12.9, 99.1)	0.131
TWiST	471.7 (388.2, 555.2)	281.6 (185.8, 377.3)	190.2 (64.2, 316.2)	0.003
REL	397.7 (310.2, 485.2)	573.6 (450.2, 697.0)	−175.9 (−324.9, −27.0)	0.021
Q‐TWiST	664.1 (613.4, 714.8)	556.5 (490.4, 622.5)	107.7 (24.9, 190.4)	0.011

*Note:* Data are restricted means at the maximum follow‐up time (1247 days). Utility values of 0.8 for TWiST, 0.6 for TOX, and 0.4 for REL were used for TWiST estimation. TOX was defined as grade 2+ TEAEs.

Abbreviations: CI, confidence interval; OS, overall survival; PFS, progression‐free survival; Q‐TWiST, quality‐adjusted TWiST; REL, relapse (period from disease progression until end of follow‐up/death); TEAE, treatment‐emergent adverse event; TOX, toxicity (sum of all periods in which patients experienced grade 2+ TEAEs during the progression‐free period); TWiST, time without symptoms or toxicities.

When the TOX state was defined by FACT‐GP5 response, TWiST was longer with ponatinib than with imatinib by 245.8 days (95% CI 106.4–385.1; *p* = 0.001) (Table 3; Figure S2). Mean TOX was 20.8 days in the ponatinib arm and 33.3 days in the imatinib (*p* = 0.377). The relative Q‐TWiST gain with ponatinib compared with imatinib was 11.59%.

**TABLE 3:** Restricted mean duration of each health state and Q‐TWiST with the TOX state defined by FACT‐GP5 response (sensitivity analysis).

Mean (95% CI) duration, days				
States	Ponatinib (*n* = 164)	Imatinib (*n* = 81)	Difference in means (95% CI), days (ponatinib vs. imatinib)	*p*
OS	1082.2 (1017.6, 1146.8)	1024.8 (918.2, 1131.4)	57.4 (−66.7, 181.5)	0.365
PFS	684.5 (592.0, 777.1)	451.2 (337.8, 564.7)	233.3 (88.7, 377.9)	0.002
TOX	20.8 (8.8, 32.8)	33.3 (8.8, 57.7)	−12.4 (−40.1, 15.2)	0.377
TWiST	663.7 (572.9, 754.5)	417.9 (309.9, 526.0)	245.8 (106.4, 385.1)	0.001
REL	397.7 (306.8, 488.7)	573.6 (448.5, 698.7)	−175.9 (−330.5, −21.4)	0.026
Q‐TWiST	702.5 (650.0, 755.1)	583.8 (514.2, 653.4)	118.8 (29.5, 208.0)	0.009

*Note:* Data are restricted means at the maximum follow‐up time (1247 days). Utility values of 0.8 for TWiST, 0.6 for TOX, and 0.4 for REL were used for TWiST estimation. TOX was defined as a FACT‐GP5 response of “quite a bit” or “very much”.

Abbreviations: CI, confidence interval; FACT‐GP5, Functional Assessment of Cancer Therapy–Leukemia item GP5 (“I am bothered by side effects of treatment”); OS, overall survival; PFS, progression‐free survival; Q‐TWiST, quality‐adjusted TWiST; REL, relapse (period from disease progression until end of follow‐up/death); TOX, toxicity (sum of all periods in which patients' FACT‐GP5 response was “quite a bit” or “very much” during the progression‐free period); TWiST, time without symptoms or toxicities.

## 4 Discussion

In this post hoc Q‐TWiST analysis using PhALLCON data, patients receiving ponatinib showed significantly longer Q‐TWiST than those receiving imatinib, and the relative Q‐TWiST gain of 10.98% was considered clinically important. In the threshold analysis, Q‐TWiST was consistently longer with ponatinib compared with imatinib, and the Q‐TWiST gain was statistically significant when the REL utility was lower than approximately 0.47. The robustness of the base‐case findings was supported by the results of sensitivity analyses using different TOX definitions. Sensitivity analyses at various follow‐up durations demonstrated increasing Q‐TWiST gains with longer follow‐up.

For patients with hematologic cancers, extending survival and preserving health‐related quality of life are important considerations [26]. Assessing the value of a treatment based on patient preferences (i.e., health utilities) regarding toxicity and clinical benefit can help inform decision‐making for patients and clinicians. The Q‐TWiST analysis has been used to assess net clinical benefits of various cancer treatments, including those for acute myeloid leukemia and chronic lymphocytic leukemia [27, 28, 29, 30]. However, to our knowledge, this is the first Q‐TWiST analysis of a treatment for ALL, providing a benchmark for future research. This analysis also adds data on quality‐adjusted survival in patients with newly diagnosed Ph+ ALL.

A systematic review of 81 Q‐TWiST comparisons between cancer treatments and control arms found a mean relative Q‐TWiST gain of 7.8% across various cancer types, with 40% of these comparisons showing a relative Q‐TWiST gain of ≥ 10% [27]. In our analysis, the relative Q‐TWiST gain of 10.98% with ponatinib versus imatinib is larger than approximately 60% of the Q‐TWiST comparisons in the systematic review [27]. This offers additional support for the clinical benefits of ponatinib in patients with newly diagnosed Ph+ ALL.

This is also the first Q‐TWiST analysis with TOX defined based on patient‐reported overall tolerability, assessed by the FACT‐GP5. The FACT‐GP5 is recommended by the US Food and Drug Administration for evaluating overall treatment tolerability from the patient perspective [31], and FACT‐GP5 scores have been shown to correlate well with overall quality of life, functional status, and clinician‐rated adverse events [32, 33, 34, 35]. As the FACT‐GP5 directly captures patients' experiences with adverse events during treatment, its use for the TOX definition may better reflect the negative impact of treatment toxicities on patients than TEAEs. In our analysis, the relative Q‐TWiST gain with ponatinib over imatinib was slightly different when TOX was defined based on the FACT‐GP5 than on clinician‐rated TEAEs (11.59% vs. 10.98%), suggesting the added value in the treatment benefits of ponatinib from patients' perspectives. Regardless of the definition used for the TOX state, time spent in TOX was not significantly different between the two arms. This is consistent with the similar safety profiles of ponatinib and imatinib in PhALLCON [9] and indicates that the longer exposure to ponatinib than to imatinib in PhALLCON did not lead to significantly more time spent with grade 2+ or grade 3+ TEAEs.

Limitations of this study include the protocol‐defined event‐free survival and OS data being immature at the data cutoff. As the absolute Q‐TWiST gain increased with follow‐up time, the observed treatment benefits of ponatinib over imatinib could have been underestimated. Additionally, the sample size is small, particularly for the imatinib arm (*n* = 81). This could result in less reliable estimates and wider CIs, and also limit the generalizability of the findings to a larger population. Therefore, the data need to be interpreted with caution, and these analyses should be revisited when mature OS data are available. Moreover, the use of last observation carried forward for handling missing FACT‐GP5 data could introduce bias. However, missing assessments were more frequent in the imatinib arm than in the ponatinib arm and are typically associated with worse PROs, as patients with TEAEs or treatment intolerance are more likely to discontinue treatment and/or skip PRO assessments. Thus, the bias could lead to an underestimation of ponatinib's benefits over imatinib. Lastly, the use of fixed utility values does not reflect the fact that utility values can vary among individuals. However, the threshold analysis provided results for various combinations of TOX and REL utilities.

## 5 Conclusion

In this post hoc Q‐TWiST analysis, ponatinib was associated with significantly and meaningfully longer quality‐adjusted survival than imatinib in patients with Ph+ ALL. The findings suggest that the superior efficacy of ponatinib over imatinib is not at the expense of patients' quality of life, further supporting the clinical benefit–risk profile of ponatinib as a front‐line therapy for patients with Ph+ ALL. This analysis provides context for risk–benefit trade‐offs between ponatinib and imatinib, facilitating shared treatment decision‐making by patients and clinicians.

## Author Contributions

**Ajibade Ashaye:** conceptualization (equal), project administration (equal), resources (lead), supervision (equal), writing – review and editing (equal). **Ling Shi:** conceptualization (equal), data curation (equal), formal analysis (lead), project administration (equal), writing – review and editing (equal). **Ibrahim Aldoss:** writing – review and editing (equal). **Pau Montesinos:** writing – review and editing (equal). **Pankit Vachhani:** writing – review and editing (equal). **Vanderson Rocha:** writing – review and editing (equal). **Cristina Papayannidis:** writing – review and editing (equal). **Jessica T. Leonard:** writing – review and editing (equal). **Maria R. Baer:** writing – review and editing (equal). **Jose‐Maria Ribera:** writing – review and editing (equal). **James McCloskey:** writing – review and editing (equal). **Jianxiang Wang:** writing – review and editing (equal). **Deepali Rane:** writing – review and editing (equal). **Shien Guo:** supervision (equal), writing – review and editing (equal).

## Ethics Statement

The trial was conducted in accordance with the International Council for Harmonization Good Clinical Practice Guideline and the principles of the Declaration of Helsinki. The study protocol and all amendments were approved by the independent institutional review board or independent ethics committee at each study site.

## Consent

All patients provided written informed consent prior to study participation.

## Conflicts of Interest

Ajibade Ashaye and Deepali Rane report employment with Takeda. Ling Shi and Shien Guo report employment with Evidera, which received payment for the statistical analysis during the conduct of the study. Ibrahim Aldoss reports honoraria from Takeda, Amgen, Pfizer, Jazz, and Sobi; consulting/advisory roles at Takeda, Amgen, Pfizer, Jazz, and Sobi; participation in the speakers bureau at Pfizer; and research funding from AbbVie and MacroGenics. Pau Montesinos reports consulting or advisory roles at Takeda, Daiichi Sankyo, and Bristol Myers Squibb; participation in the speakers bureau at Servier; and research funding from AbbVie, Takeda, Daiichi Sankyo, Novartis, and Servier. Pankit Vachhani reports consulting/advisory roles at Blueprint Medicines, AbbVie, Amgen, Cogent Biosciences, Incyte, CTI BioPharma, Daiichi Sankyo, GlaxoSmithKline, Karyopharm, Novartis, Pfizer, Genentech, Servier, Stemline, and Morphosys. Vanderson Rocha reports consulting/advisory roles at Pfizer, Takeda, AbbVie, Astellas, Kite, and Amgen. Cristina Papayannidis reports consulting/advisory roles at AbbVie, Astellas, Novartis, Bristol Myers Squibb, Menarini, Stemline, Blueprint Medicines, Incyte, GlaxoSmithKline, Amgen, Pfizer, and Janssen; and personal fees from Astellas, Novartis, Pfizer, and Amgen. Jessica T. Leonard reports consulting/advisory roles at Adaptive Biotechnologies, Kite/Gilead, Pfizer, and Takeda; and support for travel, accommodation, and expenses from Adaptive Biotechnologies. Jose‐Maria Ribera reports consulting/advisory roles at Incyte, Pfizer, Bristol Myers Squibb, Novartis, and Takeda; and research funding from Amgen. James McCloskey reports personal fees from Bristol Myers Squibb, Takeda, Blueprint Medicine, PharmaEssentia, CTI, GlaxoSmithKline, Incyte, Amgen, and Jazz Pharmaceuticals. Jianxiang Wang reports an advisory role at Abbvie. Maria R. Baer has no conflicts of interest to disclose.

## Supporting information


**Figure S1.** Partitioned survival curves in the sensitivity analysis with grade 2+ TEAEs included in the TOX state. (A) Ponatinib. (B) Imatinib. Abbreviations: REL, relapse (period from disease progression until end of follow‐up/death); TEAE, treatment‐emergent adverse event; TOX, toxicity (sum of all periods in which patients experienced grade 2+ TEAEs during the progression‐free period); TWiST, time without symptoms or toxicities.

**Figure S2.** Partitioned survival curves in the sensitivity analysis with TOX defined by FACT‐GP5 response. (A) Ponatinib. (B) Imatinib. Abbreviations: FACT‐GP5, Functional Assessment of Cancer Therapy–Leukemia item GP5 (“I am bothered by side effects of treatment”); REL, relapse (period from disease progression until end of follow‐up/death); TEAE, treatment‐emergent adverse event; TOX, toxicity (sum of all periods in which patients’ FACT‐GP5 response was “quite a bit” or “very much” during the progression‐free period); TWiST, time without symptoms or toxicities.

## Data Availability

The data sets, including the redacted study protocol, redacted statistical analysis plan, and individual participant data of the completed study supporting the results reported in this article, will be made available within 3 months from the initial request to researchers who provide a methodologically sound proposal. The data will be provided after de‐identification, in compliance with applicable privacy and data protection laws, and requirements for consent and anonymization.
