# Supplementary figures and images for: Quality‐Adjusted Time Without Symptoms of Disease or Toxicity (Q‐TWiST) in Patients With Newly Diagnosed Philadelphia Chromosome‐Positive Acute Lymphoblastic Leukemia: A Comparison of Ponatinib Versus Imatinib
Source: Cancer Med. 2025 Mar 31;14(7):e70780. doi: 10.1002/cam4.70780 (PMC11958597; doi:10.1002/cam4.70780)

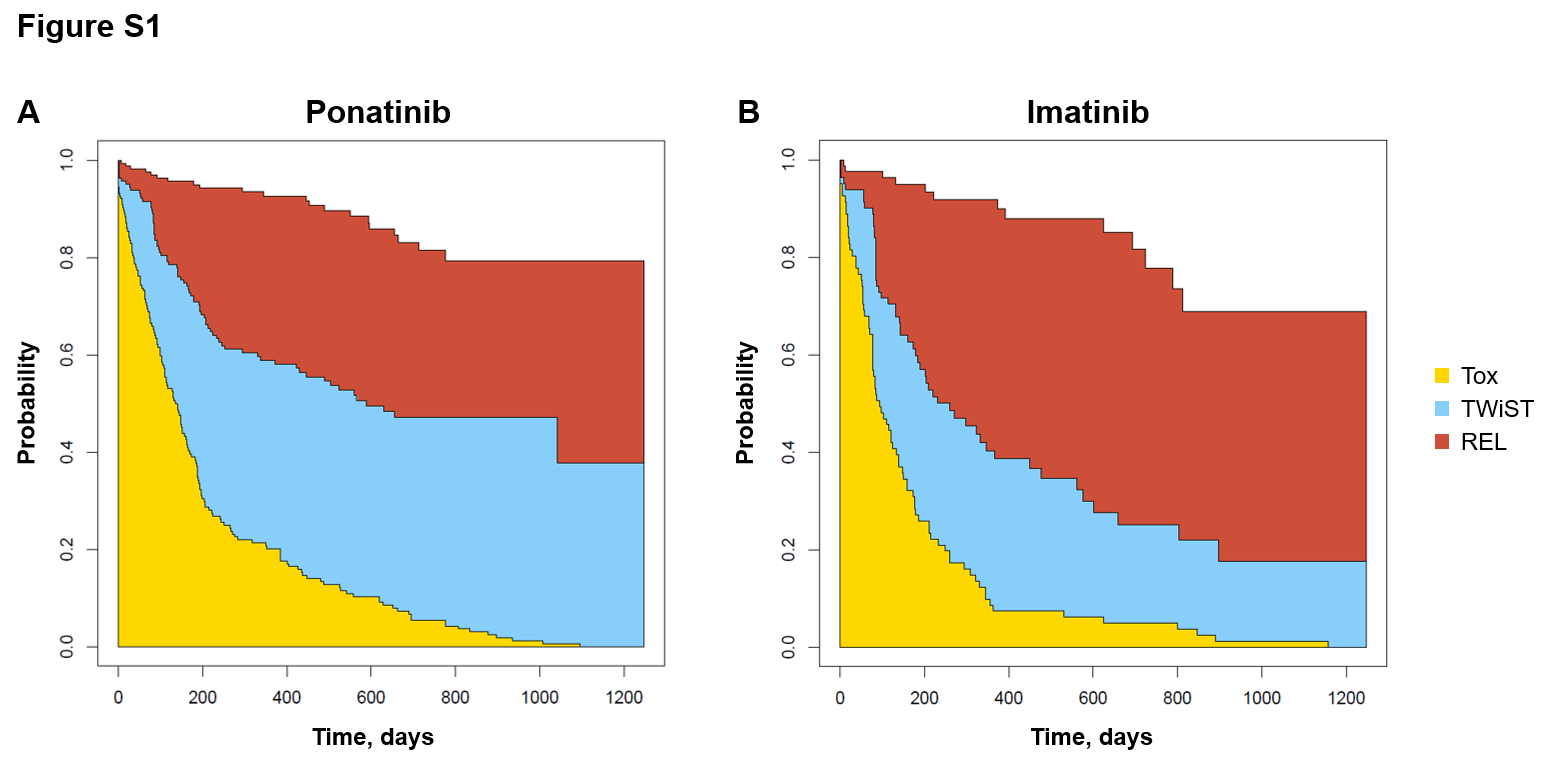

Supplement: Supplementary file 1 — Figure S1. Partitioned survival curves in the sensitivity analysis with grade 2+ TEAEs included in the TOX state. (A) Ponatinib. (B) Imatinib. Abbreviations: REL, relapse (period from disease progression until end of follow‐up/death); TEAE, treatment‐emergent adverse event; TOX, toxicity (sum of all periods in which patients experienced grade 2+ TEAEs during the progression‐free period); TWiST, time without symptoms or toxicities. [file CAM4-14-e70780-s001.tif]

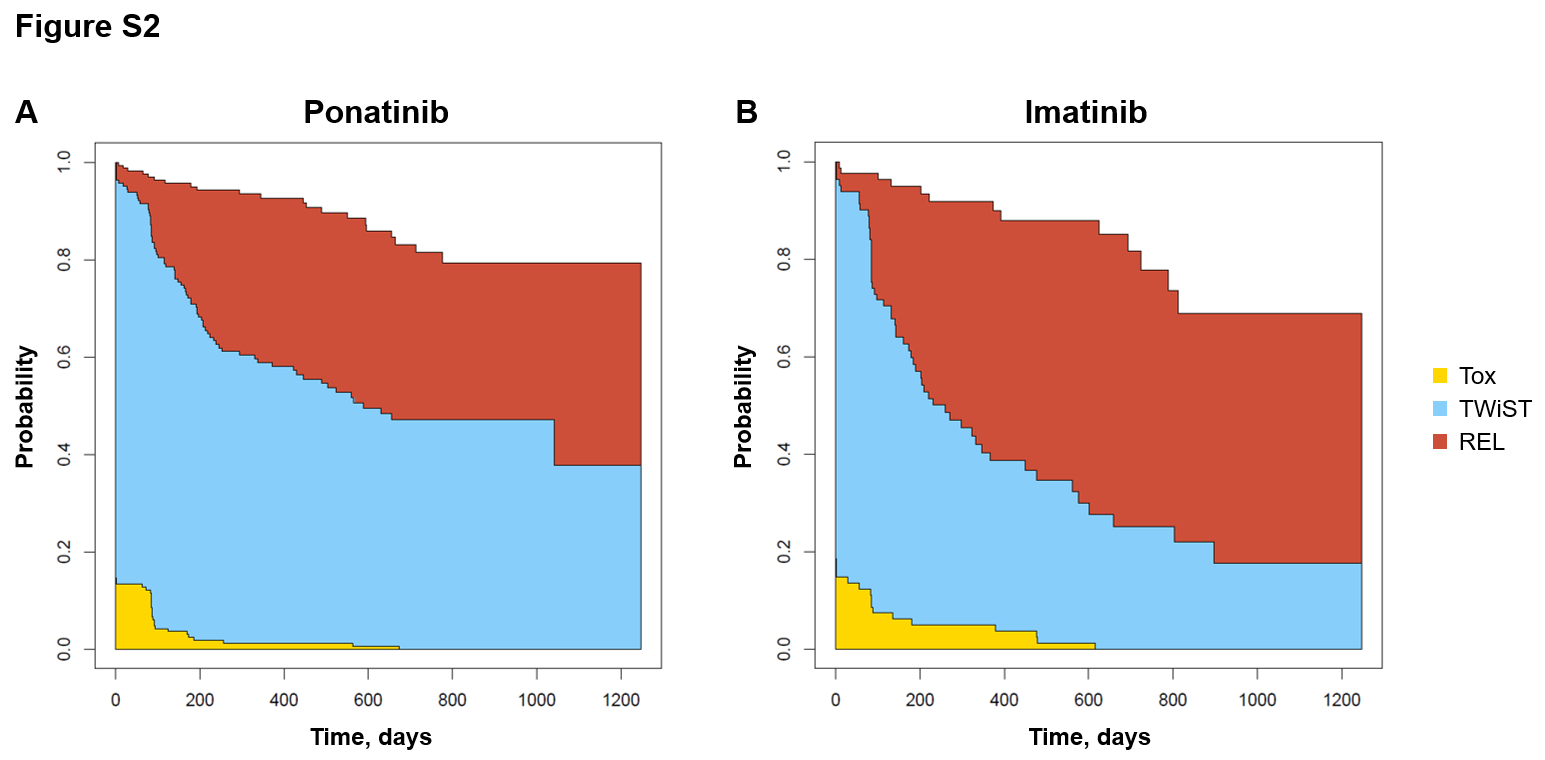

Supplement: Supplementary file 2 — Figure S2. Partitioned survival curves in the sensitivity analysis with TOX defined by FACT‐GP5 response. (A) Ponatinib. (B) Imatinib. Abbreviations: FACT‐GP5, Functional Assessment of Cancer Therapy–Leukemia item GP5 (“I am bothered by side effects of treatment”); REL, relapse (period from disease progression until end of follow‐up/death); TEAE, treatment‐emergent adverse event; TOX, toxicity (sum of all periods in which patients’ FACT‐GP5 response was “quite a bit” or “very much” during the progression‐free period); TWiST, time without symptoms or toxicities. [file CAM4-14-e70780-s002.tif]
